# Homogeneity- and Stoichiometry-Induced Electrical and Optical Properties of Cu-Se Thin Films by RF Sputtering Power

**DOI:** 10.3390/ma16186087

**Published:** 2023-09-06

**Authors:** Sara Kim, Yong-Seok Lee, Nam-Hoon Kim

**Affiliations:** Department of Electrical Engineering, Chosun University, Gwangju 61452, Republic of Korea; sarakim@chosun.kr (S.K.); ins94@hanmail.net (Y.-S.L.)

**Keywords:** Cu-Se thin films, RF magnetron sputtering, CuSe_2_ multi-component target, optical and electrical properties, stoichiometry

## Abstract

P-type Cu-Se thin films were deposited on glass substrates at room temperature using radio frequency magnetron sputtering by a single multi-component CuSe_2_ target. When using a multi-component target, the impact of the sputtering power on the homogeneity and stoichiometry within the thin films should be investigated in the depth direction to demonstrate a secondary effect on the electrical and optical properties of the thin films. Systematic characterization of the Cu-Se thin films, including the morphology, microstructure, chemical composition, and depth-directional chemical bonding state and defect structure of the thin films, revealed that the sputtering power played an important role in the homogeneity and stoichiometry of the thin films. At very low and very high sputtering power levels, the Cu-Se thin films exhibited more deviations from stoichiometry, while an optimized sputtering power resulted in more homogenous thin films with improved stoichiometry across the entire thin film thickness in the X-ray photoelectron spectroscopy depth profile, despite showing Se deficiency at all depths. A rapid decrease in carrier concentration, indicating a reduction in the net effect of total defects, was obtained at the optimized sputtering power with less deviation from stoichiometry in the Cu-Se thin films and the closest stoichiometric ratio at an intermediate depth.

## 1. Introduction

Copper selenides (Cu-Se) are important members of the first-row transition metal chalcogenides. These are a family of inorganic compounds that contain a transition metal and a chalcogen (oxygen, sulfur, selenium, or tellurium). These materials are promising candidates for a wide range of technological applications in electronics, photovoltaics, optics, photonics, optoelectronics, and thermoelectrics due to their wide range of electrical and optical properties [1]. The metal chalcogenide semiconductor Cu-Se is present in a variety of phases and crystallographic forms. It exists not only in various stoichiometric compositions, including CuSe, CuSe_2_, Cu_3_Se_2_, Cu_5_Se_4_, and Cu_7_Se_4_, but also in non-stoichiometric forms represented by CuSe_2−x_ and Cu_2−x_Se [2]. Stoichiometric CuSe_2_ refers to the ideal composition where the ratio of the Cu atoms to the Se atoms is precisely 1:2. There are two known polymorphs of CuSe_2_: marcasite and the high-pressure phase pyrite, which is metastable under ambient conditions [3]. This particular structure is characterized by interwoven face-centered cubic lattices composed of both metal cations and anion dimers, which is similar to the rocksalt structure synthesized using high-temperature solid-state reactions between Cu and Se sources [4]. On the other hand, non-stoichiometric forms of CuSe_2_ refer to compositions where the ratio of Cu-to-Se atoms deviates from the ideal 1:2 ratio. In contrast, CuSe displays a hexagonal structure with a superstructure (P63/m) that arises from the occurrence of twinning [5]. Cu_2−x_Se, which is the most extensively studied form of Cu-Se, remains the subject of controversy regarding its crystal structures and phase transition type. Within the temperature range of 350–410 K, Cu_2−x_Se undergoes a reversible phase transition between its low-temperature α-phase and high-temperature β-phase, with the exact transition temperature being contingent upon the degree of Cu deficiency present [6].

The non-stoichiometric forms can exhibit unique electrical and optical properties that differ from those of the stoichiometric form, making themselves attractive solutions for use in specific applications. The thermal stability of Cu is strongly correlated with the stoichiometric composition, as can be seen from the phase diagram of the Cu-Se system [7]. To achieve optimal performance in Cu-Se thin films for photovoltaics, it is essential to maintain a homogeneous distribution of Cu and Se atoms throughout the thickness of the thin film and minimize stoichiometry deviations during fabrication. Non-uniformity in composition can lead to variations in the electrical and optical properties of the thin film, resulting in reduced device performance. The electronic state of Cu-Se also depends on several factors, particularly including the stoichiometry. Pure Cu-Se is a p-type semiconductor with a predominance of holes (positively charged carriers) in its valence band [8,9], which is due to the intrinsic defects in the crystal lattice structure of Cu-Se creating a high concentration of acceptor states within its band gap. The distorted tetrahedral coordination geometry arises due to the Jahn–Teller effect, which is a distortion of an electronic system with degenerate energy levels that creates a lower energy state [10].

Optimum performance in Cu-Se thin films can be achieved by maintaining a uniform distribution of Cu and Se atoms throughout the thickness of the thin film and by minimizing the stoichiometry deviation during the fabrication process. To ensure compositional homogeneity and to accurately control stoichiometric deviations, precise control of the fabrication parameters that affect the growth and nucleation processes of the thin films, such as the deposition rate and temperature, is required throughout the growth of the thin films. Both the stoichiometric and non-stoichiometric forms of Cu-Se have typically been prepared by various methods, including chemical vapor deposition, electrochemical deposition, and radio frequency (RF) magnetron sputtering [1,11,12]. Among them, RF sputtering is a beneficial method of preparing Cu-Se thin films to ensure the stability and performance of the thin films due to its advantages, which include precise control over the film properties, a fast deposition rate, and excellent adhesion to substrates. The latter is particularly important in ensuring the stability and performance of the Cu-Se thin films. To produce high-quality single-phase Cu-Se thin films, it is necessary to optimize the process parameters, such as the sputtering power. Nonetheless, the effect of the sputtering power on the properties and structure of deposited films has rarely been investigated [13], and there is a paucity of research on this topic for Cu-Se in the academic literature. In this study, Cu-Se thin films were prepared by a one-step RF magnetron sputtering technique at ambient temperature in order to demonstrate the effect of the sputtering power on the film homogeneity in the depth direction and its secondary effects on the electrical and optical properties of the thin films. Then, an optimization process was carried out to identify the ideal sputtering power conditions that could fabricate the Cu-Se thin films with superior homogeneity and stoichiometry by investigating the effect of the sputtering power on the morphology, microstructure, chemical composition, depth-directional chemical bonding state and defect structure, and electrical/optical properties of the thin films, to improve their quality.

## 2. Experimental Details

Experiments were designed to determine the optimum and precise conditions for the deposition of Cu-Se thin films by using the one-step RF magnetron sputtering technique on 1 cm × 1 cm Corning glass substrates. The sputtering process employed an RF magnetron sputtering system (IDT Engineering Co., Gyeonggi, Republic of Korea) with a CuSe_2_ target (TASCO, Seoul, Republic of Korea, 99.99% purity, 2-inch diameter). The sputtering power was varied from 20 to 100 W in 20 W increments, while the other sputtering parameters were kept constant, as tabulated in Table 1. The glass substrates were cleaned ultrasonically using acetone, alcohol, and deionized water sequentially. During the target cleaning process, a shutter was used to shield the substrates. The deposition time was adjusted to achieve a uniform thickness of approximately 400 nm for the accurate comparison of each specimen. X-ray diffraction (XRD, PANalytical B.V., Almelo, The Netherlands, X’pert-PRO-MRD, Cu Kα = 0.15405 nm, 40 kV, 30 mA) analysis was carried out to examine the crystallographic structure of the Cu-Se thin films over a 2*θ* range of 10–90° with a step size of 0.026° and scanning speed of 8.5°/min. The morphological characteristics of the thin films were studied using a field emission scanning electron microscope (FESEM, Hitachi, Tokyo, Japan, S-4700, without Pt coating) and an atomic force microscope (AFM, NT-MDT Inc., Moscow, Russia, NTEGRA Prima System, a typical tip curvature radius of 8–10 nm) at room temperature and under ambient conditions. The compositional analysis and chemical nature of the Cu-Se thin films were achieved using an energy-dispersive X-ray spectrometer (EDS) attached to the FESEM and an X-ray photoelectron spectroscope (XPS, Thermo Fisher Scientific Inc., Waltham, MA, USA, K-Alpha^+^). The optical properties of the thin films were measured using a UV–visible spectrophotometer (Varian Techtron, Mulgrave, Australia, Cary500scan) in the range of 200–3000 nm. The electrical properties, including the carrier concentration, resistivity, and mobility of the films, were determined using a Hall effect measurement system (Accent Optical Technologies Inc., Bend, OR, USA, HL5500PC) at room temperature.

## 3. Results and Discussion

**Figure 1:**
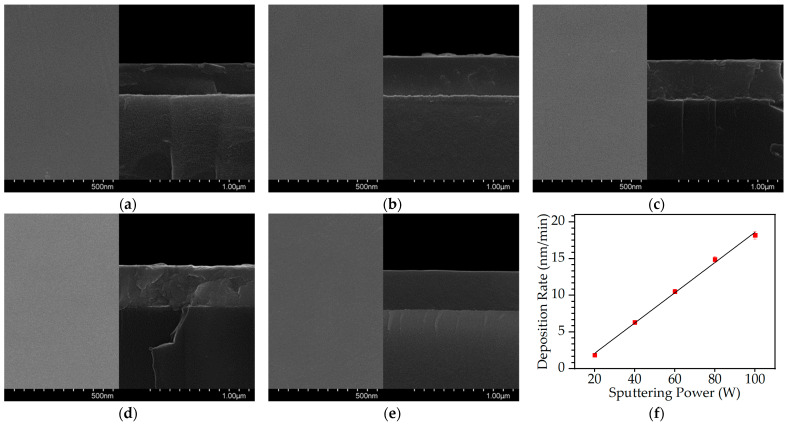
Surface and cross-sectional field emission scanning electron microscope (FESEM) images of Cu-Se thin films with different sputtering powers of (**a**) 20, (**b**) 40, (**c**) 60, (**d**) 80, and (**e**) 100 W. (**f**) Deposition rates of the Cu-Se thin films as a function of sputtering power with a linear trend line.

Surface and cross-sectional FESEM images of the deposited Cu-Se films with varying sputtering power are shown in Figure 1. All of the thin films exhibited a uniform amorphous structure with no discernible grain boundaries or crystallites. FESEM surface images of each specimen revealed a smooth and featureless morphology with no significant changes in film morphology regardless of the sputtering power used. Although increasing the sputtering power resulted in a noticeable increase in the deposition rate, it did not significantly affect the amorphous structure of the thin films, such that the thin films showed no grain boundaries even at high sputtering powers up to 100 W. This suggests that the surface topography and grain morphology of the Cu-Se thin films were not significantly affected by the sputtering power used in the deposition process. The deposition rates were 1.87, 6.3, 10.5, 14.89, and 18.2 nm/min at the sputtering powers of 20, 40, 60, 80, and 100 W, respectively, as shown in Figure 1f. The deposition rate of the thin films increased almost linearly with increasing sputtering power, which indicates that the number of sputtered particles is proportional to the number of ions or neutral particles that collide with the target surface and can be attributed to the direct influence of the sputtering power on the kinetic energy of the sputtered particles. A common feature of sputtering, which can be used to tune the deposition rate of thin films in many technological applications, is the increase in deposition rate with increasing sputtering power [14]. As the sputtering power is increased, the kinetic energy of the sputtered particles increases, resulting in a higher momentum and consequently an increase in deposition rate. It should be noted that, however, although a linear correlation between the sputtering power and the deposition rate has been established, the validity of this relationship may be restricted to a certain range of power values. This is because the dependence would not be linear when two factors influence the deposition rate, and excessively high levels of sputtering power have the potential to induce plasma instabilities, which may have unexpected effects on the deposition process [15].

**Figure 2:**
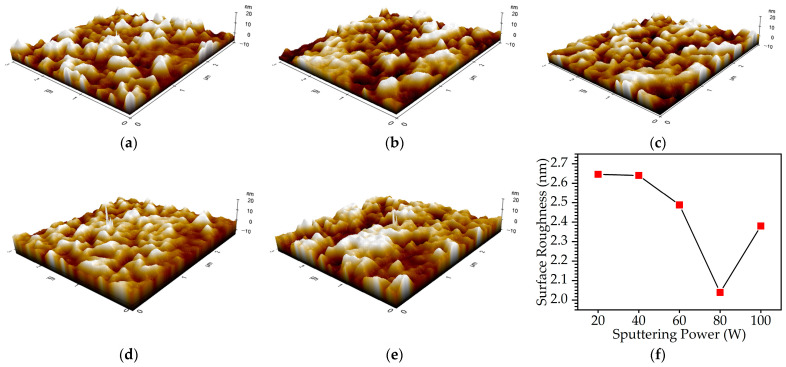
Atomic force microscopy (AFM) image of Cu-Se thin films covering an area of 3 µm × 3 µm with different sputtering powers of (**a**) 20, (**b**) 40, (**c**) 60, (**d**) 80, and (**e**) 100 W. (**f**) Average roughness (R_a_) of the Cu-Se thin films under the sputtering power variations.

AFM was employed to analyze the surface morphology of the Cu-Se thin films deposited on a glass substrate by the sputtering method. Figure 2a–e show the three-dimensional views of the Cu-Se thin films deposited on glass substrates, covering an area of 3 µm × 3 µm. The AFM topography indicated that the Cu-Se thin films were uniform, and no grains were observed, in agreement with the FESEM results. The average roughness (R_a_) was utilized as a quantitative measure to evaluate the surface roughness of the Cu-Se thin films. The surface roughness in Cu-Se thin films arises as a natural outcome of their three-dimensional growth process and plays a crucial role in influencing the performance of optical devices employing these films. The roughness acts as a scattering interface for electron propagation and additionally impacts the behavior of electromagnetic waves [16]. For the Cu-Se thin films, the obtained R_a_ values were 2.646, 2.640, 2.488, 2.039, and 2.38 nm for sputtering powers of 20, 40, 60, 80, and 100 W, respectively. The AFM results demonstrated a non-linear trend in the surface roughness as a function of the sputtering power, as shown in Figure 2f. Initially, as the sputtering power increased from 20 to 80 W, the R_a_ values exhibited a decreasing trend, indicative of smoother surfaces. This trend can be attributed to enhanced sputtering efficiency and improved surface coverage, resulting in a reduction in surface irregularities and defects [17]. However, beyond the 80 W threshold, the trend was reversed, with the average roughness increasing as the sputtering power was further elevated from 80 to 100 W. This counterintuitive behavior is likely due to heightened ion bombardment effects and excessive thermal generation at higher sputtering powers [18].

**Figure 3:**
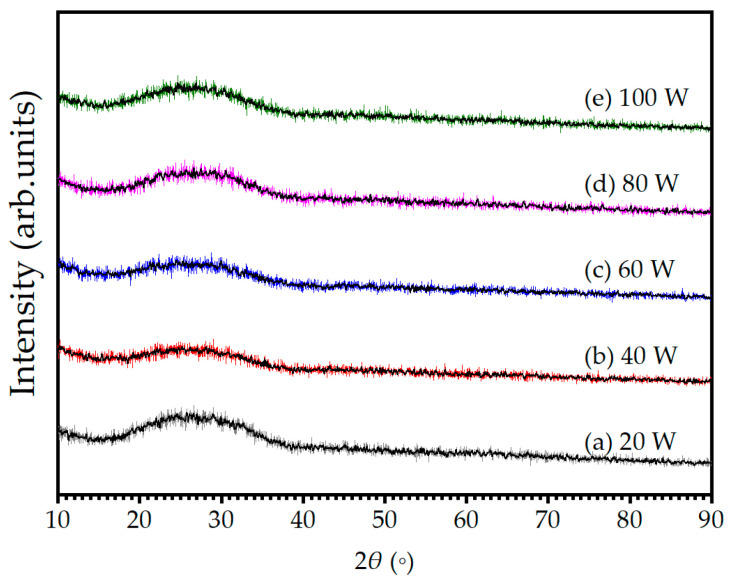
X-ray diffraction (XRD) patterns of as-deposited Cu-Se thin films with different sputtering powers of (**a**) 20, (**b**) 40, (**c**) 60, (**d**) 80, and (**e**) 100 W.

Figure 3 exhibits the XRD patterns of Cu-Se thin films with different sputtering powers, which showed a broad halo without any sharp diffraction peaks in all specimens. The absence of any distinct peaks in the XRD pattern is attributed to the lack of long-range order in the amorphous structure, where the atoms were arranged in a disordered manner. Furthermore, the broad halo in the XRD pattern suggested that the atoms in the amorphous Cu-Se thin films were arranged in a relatively disordered fashion at the short-range scale, which is in contrast to the ordered arrangement of atoms in a crystalline material. Overall, the XRD pattern analysis confirms the amorphous nature of the Cu-Se thin film. It is worth noting that the absence of peaks in the XRD patterns does not necessarily mean that the thin films are of poor quality or lack useful properties. Amorphous or nanocrystalline materials can have desirable properties such as a large surface area, enhanced catalytic activity, and improved mechanical properties. However, if the intended applications of the Cu-Se thin films require a precise crystal structure or orientation, additional processes such as annealing or other related techniques may be indispensable to achieve the desired result.

**Figure 4:**
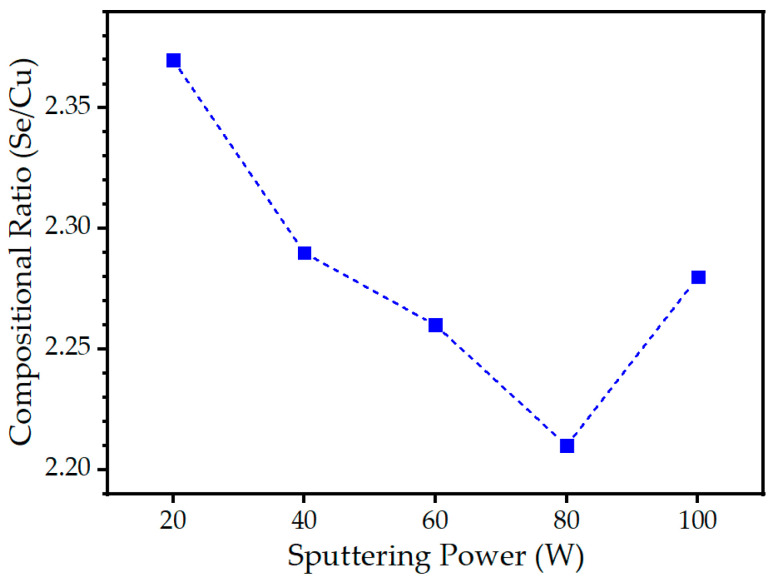
Energy-dispersive X-ray spectrometry (EDS) compositional ratio (Se/Cu) of the Cu-Se thin films with different sputtering powers of 20, 40, 60, 80, and 100 W.

The effect of sputtering power on the chemical composition of Cu-Se thin films was investigated using EDS analysis. The EDS analyses of the as-deposited thin films indicated Se-rich compositions in comparison to the stoichiometric ratio, when the sputtering target had a stoichiometric Se to Cu ratio of 2.00. Figure 4 shows the variation in the Se/Cu compositional ratio in the Cu-Se thin films with different sputtering powers. The Se/Cu compositional ratio in the Cu-Se thin films exhibited a decreasing trend as the sputtering power increased from 20 to 80 W, with values of 2.37, 2.29, 2.26, and 2.21, before increasing again to 2.28 at a higher sputtering power of 100 W. The Cu-Se thin film deposited at 80 W sputtering power showed the compositional ratio closest to the stoichiometric ratio of 1:2 among all the as-deposited Cu-Se thin films. In general, increasing the sputtering power led to an increase in the thickness of the deposited films, i.e., the deposition ratio, as a result of the increased number of atoms ejected from the target material; however, the increase in Cu and Se may not be proportional, leading to changes in the Cu/Se compositional ratio. The sputtering yield, which assumes that the ion–target interaction in the binary collision approximation (BCA) model can be approximated as a series of separate interactions between the ion’s kinetic energy and the mass of the target atoms, for each Cu and Se atom can also lead to a change in the initial composition of the multi-component CuSe_2_ sputtering target. However, this change is balanced by the fact that the concentration of elements preferentially sputtered on the surface will decrease to achieve an equilibrium composition at the target surface [19], typically after the first ten nanometers of deposition. If the sputtering yields are significantly different from each other, the equilibrium composition can drift away from the stoichiometry, resulting in a loss of stoichiometry in the deposited film compared to the target material [19]. However, the different sputtering yield for each element is not sufficient to explain the overall Se-rich composition under all sputtering power conditions and the increase in the Se/Cu compositional ratio again at 100 W, as shown in Figure 4. In the context of sputter deposition, the interaction between the target material and the substrate involves certain fundamental processes. The primary processes that occur between the target and the substrate are scattering and differential scattering, whereby the elemental ratio in the substrate film can be altered by the scattering of the constituent elements in the multi-component target. At the substrate level, elemental loss can occur in various forms, including re-sputtering, incomplete adhesion, diffusion, and re-evaporation [20]. Under conditions of comparable values of the mean free path and the substrate-to-target distance, the difference in the scattering of various sputtered species can lead to the alteration of the composition of deposited films [21]. In the context of the present experimental study, as previously elucidated, the Se/Cu compositional ratio that most closely approximated the stoichiometric value of 2.0 was achieved under the optimized sputtering condition of 80 W. However, subsequent increments in the sputtering power exhibited a discernible increase in deviation from the desired 2.0 ratio. To comprehensively explicate the underlying drivers of this phenomenon, it is imperative to consider several influential factors that could potentially contribute to further deviations in the Se/Cu compositional ratio from the ideal stoichiometric proportion. At higher sputtering power, the momentum and energy of the bombarding ions increase, leading to enhanced sputtering rates. This heightened sputtering intensity can promote the increased scattering and differential scattering of elements from the target material. Additionally, at higher sputtering power, the probability of collision events between ions and target atoms rises. These collisions can induce the preferential ejection of certain elements over others, further influencing the compositional variation in the sputtered material. The correlation between the sputtering power and the Cu/Se ratio is intricate and influenced by various factors, including the composition of the target material, deposition conditions, substrate properties, and energy distribution of the sputtered species [22], some of which were discussed above. Merely relying on the EDS results to examine the film’s composition may not provide sufficient insight. EDS offers an average atomic ratio over the analyzed volume, lacking detailed information about the atomic distribution within the film, particularly as the sputtering power varies. To gain a comprehensive understanding of the atomic distribution within the film under different sputtering power levels, additional analyses, such as depth profiling, must be conducted.

**Figure 5:**
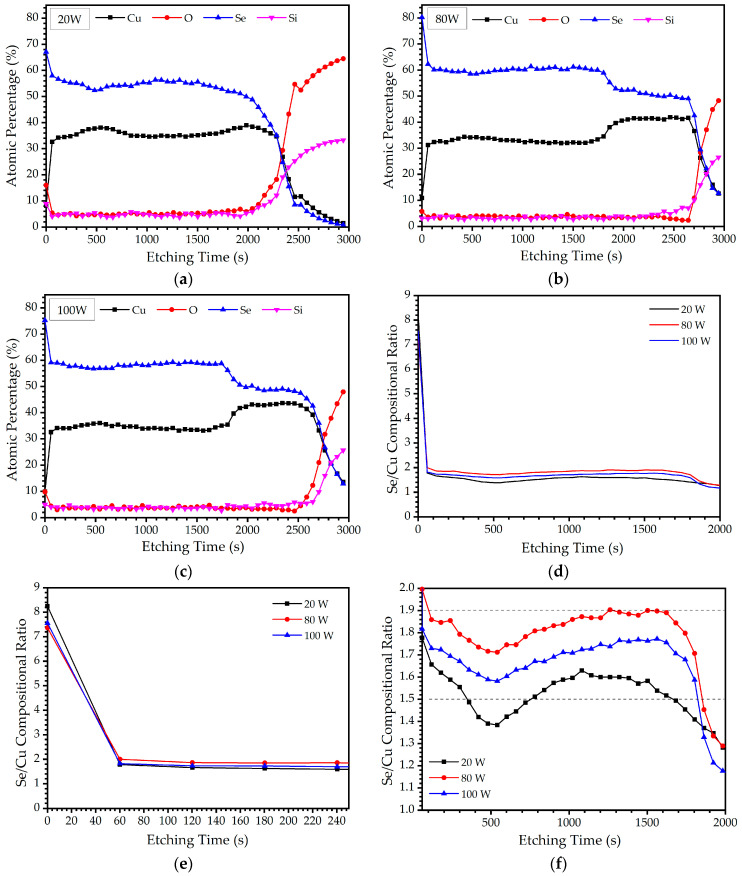
XPS depth profiles of the Cu-Se thin films with different sputtering powers of (**a**) 20, (**b**) 80, and (**c**) 100 W. Se/Cu compositional ratio profiles for the Cu-Se thin films with different sputtering powers of 20, 80, and 100 W at an etching time range of (**d**) 0–2000, (**e**) 0–250, and (**f**) 100–2000 s.

When performing thin film analysis, it is imperative to account for the compositional ratio from the substrate to the surface, as the elemental composition can exhibit significant variations at different depths within the film [23,24,25]. The XPS depth profile was analyzed to obtain a more detailed characterization of the chemical composition and distribution within the Cu-Se thin films, allowing a more accurate assessment of their properties and performance. The Cu-Se thin films were etched by Ar sputtering for 60 s, which was sequentially repeated 50 times (3000 s in total) to conduct the XPS analysis in the depth direction for all the samples. The XPS depth profile results are presented in Figure 5. The surfaces of all the Cu-Se thin films showed a higher Se/Cu compositional ratio, indicating an excess of Se at the surface. However, the Se/Cu compositional ratio decreased rapidly with an increase in the etching time to 60 s, suggesting a non-stoichiometric composition and Se deficiency in the inner film. For the Cu-Se thin films at 20 W, the Se/Cu compositional ratio decreased from 8.2 at the surface to 1.5 at an intermediate depth and further dropped to 1.0 near the glass substrate. Similarly, the Cu-Se thin films at 80 W exhibited a reduction in the Se/Cu compositional ratio from 7.3 at the surface to 1.8 in the intermediate depth and 1.1 near the substrate. The Cu-Se thin films at 100 W showed a decrease in the Se/Cu compositional ratio from 7.5 at the surface to 1.7 at an intermediate depth and subsequently to 1.1 near the substrate. It is noteworthy that the Cu-Se thin films deposited at 80 W displayed the most balanced composition throughout their depth. Across all sputtering power conditions, the conspicuous accumulation of Se was observed on and near the film surface. This phenomenon is attributed to the diffusion of Se from the film’s interior towards the surface and the vicinity of the surface. In all of the Cu-Se thin films, Se migration towards the surface of the film was observed owing to the high volatility of Se. The sharp decrease in the Se/Cu compositional ratio at the etching times between 250 and 650 s is also suggestive of Se migration towards the surface of the Cu-Se thin films. This migration is more pronounced in cases of low sputtering power (20 W), and then decreases as the sputtering power is increased up to 80 W. However, with further increases in sputtering power to 100 W, the Se migration increases again. The higher migration of Se atoms towards the surfaces of the Cu-Se thin films at 20 W can be attributed to their lower surface binding energy at lower sputtering powers. This is consistent with previous studies that have reported the higher surface migration of atoms with lower surface binding energy under similar conditions [26]. The lower migration of Se atoms towards the surfaces of the thin films at higher sputtering powers (80 and 100 W) could be due to an increase in the energy of the sputtered species, leading to stronger bonding and reduced surface mobility. Based on the observed alterations in elemental composition across the thickness of the Cu-Se thin films, particularly near the substrate, it is evident that Cu consistently demonstrates a higher deposition rate than Se during the initial stages of the sputtering process. This observation is supported by the Se/Cu compositional ratio profiles depicted in Figure 5a–c throughout the depth of the thin films. This occurrence was attributed to the higher sputtering yield of Cu from the target surface during the initial stages of sputtering, resulting in a deficiency of Se and the enrichment of Cu at the interface with the substrate. As the sputtering process continued, the temperature at the surface of the target increased due to the Ar^+^ plasma’s impact, and the sputtering yield of Se increased more through the sublimation of Se with the low surface binding energy. Meanwhile, the transportation process to the substrate was less affected because there were fewer changes in trajectory and energy loss from the collisions owing to their relatively large mass compared to Cu atoms [27], although more Se atoms were detached from the target as the sputtering process continued in the same sputtering power. These eventually led to an equilibrium condition where the Se/Cu compositional ratio stabilized at a certain value. The confirmation of this explanation is supported by the Se/Cu compositional ratio observed at an intermediate depth in the thin film, as depicted in Figure 5a–c. While the sputtering power does not have a significant impact on the number of elements that eventually reach the substrate [27], the overall higher Se/Cu compositional ratio at 80 W than at 100 W can be explained by the greater number of Cu atoms detached during the sputtering process at 80 W. These large numbers of detached Cu atoms, which have a relatively low mass in the trajectory and significant energy loss due to frequent collisions, result in a higher Se/Cu compositional ratio, according to Figure 5f. A notable disparity is observed between the results obtained from EDS and XPS analyses for the Cu-Se thin films. EDS analysis led to the conclusion that all films exhibit Se-rich characteristics. Conversely, the earlier mentioned XPS findings indicated the surface and near-surface accumulation of Se; however, the depth profile revealed a Se-poor compound across the surface, considering the desired stoichiometry of Cu-to-Se at 1:2. It is essential to recognize that XPS and EDS offer distinct depths of measurement and can therefore give different results, particularly when compositional variations are present within the material. XPS provides information primarily from very superficial layers, approximately 1 nm in depth, while EDS penetrates considerably deeper into the material, reaching depths of several micrometers, thus permitting bulk composition assessment [28,29].

**Figure 6:**
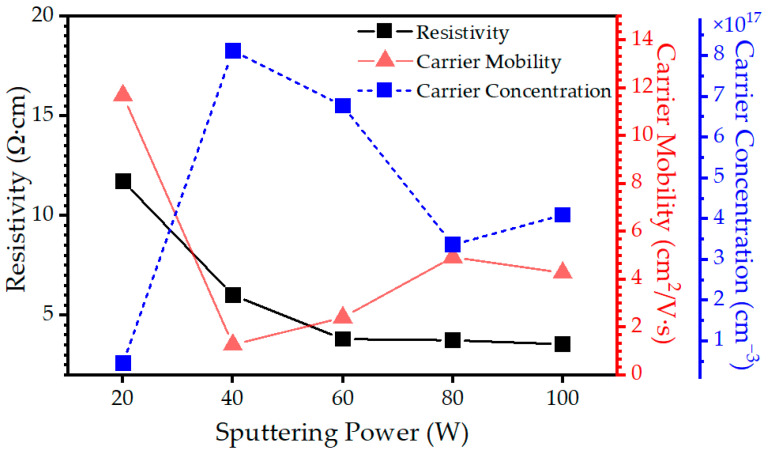
Carrier concentration (*n*), carrier mobility (*μ*), and resistivity (*ρ*) in as-deposited Cu-Se thin films as a function of sputtering power.

The electrical properties of the Cu-Se thin films were investigated using Hall effect measurements to determine the resistivity (*ρ*), carrier mobility (*μ*), and carrier concentration (*n*). The conductivity type and concentration of defects present in the Cu-Se thin films can significantly affect their electrical and optical properties. Therefore, understanding the nature and distribution of defects is critical in optimizing the performance of Cu-Se thin film-based devices. It is worth mentioning that when measuring the Hall effect, the carrier concentration typically reflects the bulk properties of the material being measured, including both the surface and the depth of the film. All the Cu-Se thin films exhibited p-type conductivity, despite demonstrating Se deficiency throughout their thickness. This behavior is consistent with typical Cu-deficient systems, which give rise to the formation of Cu vacancies, contributing to the observed p-type conductivity. A comprehensive understanding of the p-type conductivity in these Se-poor (Cu-rich) films necessitates a meticulous examination of the Cu-Se phase diagram. The analysis of the Cu-Se phase diagram reveals that when the Se atomic percentage falls within the range of 50–65% [6], the most stable phase at normal temperatures is CuSe. As a result, when the sputtering power was set at 20 W, the atomic percentages of Cu and Se indicated that, except for the surface region, Se-rich CuSe compounds were formed throughout the entire film thickness, with nearly stoichiometric CuSe observed a few nanometers above the glass substrate. On the other hand, when the sputtering power was increased to 80 W, the XPS depth profile results showed that the Cu-Se thin film exhibited Se-poor CuSe_2_ in both the surface region and intermediate depth, but not at the extreme surface due to the diffusion of Se, while showing almost Cu-poor CuSe a few tens of nanometers above the glass substrate. Similarly, for sputtering power of 100 W, the Cu-Se thin film displayed even more Se-poor CuSe_2_ in the surface region and intermediate depth compared to the 80 W case, with nearly Cu-poor CuSe observed a few tens of nanometers above the glass substrate, similar to what was observed at 80 W. In the studied systems, the Cu-Se thin films exhibited two distinct compositions, CuSe and CuSe_2_, each with varying stoichiometry. When the system had a deficiency of Cu atoms, leading to Cu-poor CuSe, vacant sites emerged within the crystal lattice where some Cu atoms were missing. These vacancies caused the formation of holes in the lattice, contributing to p-type conductivity. On the other hand, in the CuSe_2_ phase with a deficiency of Se, it introduced extra free electrons to the crystal, increasing the electron concentration. As the system consisted of a mixture of both compounds with varying defect concentrations, the overall effect on the carrier concentration depended on the relative abundance of these defects. When the concentration of Cu vacancies in the Cu-poor CuSe phase surpassed the concentration of Se vacancies in the Se-poor CuSe_2_ phase, an increase in the hole concentration occurred, resulting in p-type behavior. This observation suggests that, despite differences in film thickness and diverse structural features, the dominant contribution to p-type conductivity is driven by defects favoring hole donation over other types of defects. The carrier concentration of the Cu-Se thin films is illustrated in Figure 6. The relatively high carrier concentration in all the Cu-Se thin films is attributed to a doping effect caused by defects [30]. A noticeable decrease in the carrier concentration can be observed as the sputtering power is increased from 40 to 80 W, indicating a reduction in the net effect of electron donors and holes. The depth profile analysis supports this finding, revealing less variation in the stoichiometry of the Cu-Se thin films and the closest stoichiometric ratio at an intermediate depth. However, when the sputtering power is further increased to 100 W, the variation is amplified and a greater deviation from stoichiometry is observed, leading to a higher net effect of total defects and, consequently, an increase in the carrier concentration. As for the Cu-Se thin film at 20 W, its low carrier concentration can be attributed to its smaller thickness compared to the other thin films, resulting in the lowest net effect of different defects in the system. The carrier mobility, as shown in Figure 6, demonstrates a clear inverse relationship with the carrier concentration, indicating that as the carrier concentration increases, the carrier mobility decreases. This reduction in mobility can be attributed to various scattering mechanisms, including Coulomb scattering from impurities, surface scattering, and phonon scattering [31]. Specifically, at low-to-moderate doping concentrations, the presence of Coulombic traps generated by dopant ions is expected to impede the motion of charge carriers, resulting in a decrease in carrier mobility [32,33]. The electrical resistivity of the Cu-Se thin films is primarily determined by the carrier concentration and mobility and is related to these factors through the equation *ρ* = 1/*nqµ*, where *ρ* represents resistivity, *n* represents the carrier concentration, *q* represents the unit carrier charge, and *µ* represents carrier mobility. The electrical resistivity of the sputtered Cu-Se thin films is influenced by the interplay between carrier mobility and the carrier concentration, both of which can be affected by the amorphous structure of the materials. Higher carrier mobility and carrier concentrations usually result in lower resistivity, whereas lower mobility and carrier concentrations lead to higher resistivity. Ultimately, the electronic properties of Cu-rich CuSe and Se-poor CuSe_2_ differ, resulting in differences in their electrical resistance.

**Figure 7:**
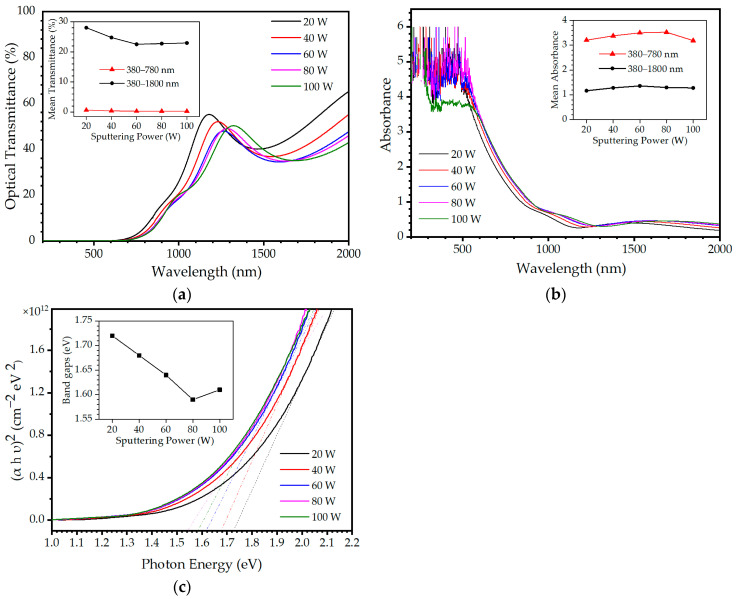
Optical properties of the Cu-Se thin films with increasing sputtering power: (**a**) optical transmittance and (**b**) absorbance in the visible and near-infrared (NIR) spectral regions. (**c**) Tauc plots for determination of the band gaps (*E_g_*) of the Cu-Se thin films using linear extrapolation of the leading edge in the direct band gap using (*αhυ*)^2^. The insets of (**a**–**c**) show the mean optical transmittance, mean absorbance, and band gap (*E_g_*) of the Cu-Se thin films, respectively.

The optical properties of the Cu-Se thin films were investigated with respect to changes in sputtering power over a wide spectral region ranging from 200 to 2000 nm. The optical transmittance of the Cu-Se thin films exhibited absorption characteristics in the visible and near infrared (NIR) spectral regions, with absorption edges at approximately 700 nm, as shown in Figure 7a. This exhibits a relatively sharp transmittance onset as a typical characteristic of direct band-gap semiconductors. A noticeable trend was observed in the absorption edge of the Cu-Se thin films as an increase in the sputtering power, wherein the edge shifted towards longer wavelengths (the Burstein–Moss shift). This shift in the absorption edge indicates that the band gap of the Cu-Se thin film decreases as the sputtering power increases. With an increase in the sputtering power in the visible spectral region 380–780 nm, the mean transmittance of the Cu-Se thin films was found to converge to a much lower value, which means that the Cu-Se thin films exhibited better optical capabilities as an absorber layer, with the mean transmittance values of 0.64, 0.38, 0.28, 0.26, and 0.25% for sputtering powers of 20, 40, 60, 80, and 100 W, respectively. The absorbance of the Cu-Se thin films is presented in Figure 7b. Absorbance is defined as the extent to which a material absorbs light, using the equation *A* = −log*T* = log(*I*_0_/*I*), where *T* is the optical transmittance, *I* is the intensity of the transmitted radiation, and *I*_0_ is the intensity of the incident radiation. The mean values of the absorbance over the 380–780 nm (visible) and 380–1800 nm (visible to NIR) spectral ranges are shown in the inset of Figure 7b. Within the visible spectral region, the mean absorbance values of the as-deposited Cu-Se thin films are 3.20, 3.38, 3.50, 3.52, and 3.18 for sputtering powers of 20, 40, 60, 80, and 100 W, respectively, while these values decrease to 1.16, 1.28, 1.36, 1.29, and 1.28 for the same powers in the visible to NIR spectral range. This means that 93.082–95.635% of the incident light is absorbed in the visible to NIR region, while 99.934–99.970% of the incident light in the visible region is absorbed by the 400-nm Cu-Se thin film. This represents very strong absorption characteristics in the visible spectral region, where it is also observed that the absorbance of the Cu-Se thin films increases with increasing sputtering power. The absorption coefficient (*α*) was calculated using the Beer–Lambert law in the high-absorption region *α*(*υ*) = (2.303*A*/*d*), where *A* is the absorbance, *υ* is the frequency, and *d* is the thickness of the thin film. The results indicate that the absorption coefficient increases with an increase in the sputtering power, which is in agreement with the observed increase in absorbance. These findings suggest that the optical properties of Cu-Se thin films are closely related to the sputtering power used during their deposition. To determine the optical band gap and the nature of optical transitions in the Cu-Se thin films, it is possible to analyze the absorption coefficient as a function of photon energy. In this context, the optical band gap of the Cu-Se thin films can be estimated by plotting the square of the product of the absorption coefficient and photon energy, i.e., Tauc’s equation (*αhν*)^2^ for direct-transition semiconductors, as a function of photon energy, *hν*. By extrapolating the linear portion of the curve that corresponds to the onset of the absorption edge to the energy axis, the band gaps of the Cu-Se thin films were estimated, as plotted in Figure 7c. The estimated band gaps of the Cu-Se thin films are 1.72, 1.68, 1.64, 1.59, and 1.61 eV for sputtering powers of 20, 40, 60, 80, and 100 W, respectively, as shown in the inset of Figure 7c. The observed trends of the band gap in Figure 7c and the resistivity in Figure 6 show striking similarity. This correlation can be explained by considering the influence of the band gap on the electrical conductivity of a material. In materials with larger band gaps, a substantial energy barrier exists between the valence and conduction bands, which hinders the transition of electrons from the valence band to the conduction band. Conversely, in materials with smaller band gaps, the energy barrier between the valence and conduction bands is diminished, enabling electrons to readily move from the valence band to the conduction band, even at lower energy levels. Consequently, these materials demonstrate enhanced electrical conductivity and exhibit lower resistivity [34,35].

## 4. Conclusions

The homogeneity across the depth was investigated in terms of the correlation with the electronic and optical properties of Cu-Se thin films with varying sputtering power. XPS depth profiling was conducted to reveal significant differences in properties, in addition to deposition rates, among films with different sputtering power, including variations in the Se/Cu compositional ratio and the presence of Cu-poor CuSe and Se-poor CuSe_2_ compounds to different degrees. The Cu-Se thin film deposited with lower sputtering power demonstrated the highest deviation from stoichiometry, while the Cu-Se thin film deposited with a relatively high sputtering power exhibited the highest homogeneity, with less deviation from stoichiometry across the depth of the film. Increasing the sputtering power beyond a certain point led to more deviation towards stoichiometry, indicating saturation in the sputtering power. The compositions of the target and the Cu-Se thin films were governed by the sputtering power, and the optimization of the sputtering power for required stoichiometry is crucial. The EDS and XPS showed significantly different results, suggesting that EDS cannot be relied on for the design of thin films. Through XPS depth profile analysis, it was demonstrated that the film homogeneity along the depth direction is affected by the sputtering power when employing multi-component targets in the sputtering process. Furthermore, the homogeneity of the film across the depth, as well as the composition of the entire thin film, should be considered in designing thin film devices for various electronics, photovoltaics, and optoelectronics.

## Figures and Tables

**Table 1:** List of sputtering parameters for the Cu-Se thin films.

Sputtering Parameter	Value
RF Sputtering Power	20/40/60/80/100 W
Base Pressure	1.0 × 10^−6^ Torr
Vacuum Pressure	7.5 × 10^−3^ Torr
Ar Gas Flow	20 sccm
Pre-Sputtering Time	5 min
Substrate-to-Target Distance	5.0 cm
Rotating Substrate Holder	11 rpm
Temperature	Room Temperature (25 °C)

## Data Availability

Data are contained within the article.
